# Effects of Dietary Protein Concentration on Lipid Metabolism Gene Expression and Fatty Acid Composition in 18–23-Month-Old Hanwoo Steers

**DOI:** 10.3390/ani11123378

**Published:** 2021-11-25

**Authors:** Rajaraman Bharanidharan, Krishnaraj Thirugnanasambantham, Ridha Ibidhi, Geumhwi Bang, Sun Sik Jang, Youl Chang Baek, Kyoung Hoon Kim, Yea Hwang Moon

**Affiliations:** 1 Department of Agricultural Biotechnology, College of Agriculture and Life Sciences, Seoul National University, Seoul 08826, Korea; bharanidharan7@snu.ac.kr; 2 Department of Eco-Friendly Livestock Science, Institute of Green Bio Science and Technology, Seoul National University, Pyeongchang 25354, Korea; thiru_dna@yahoo.co.in (K.T.); ridha@snu.ac.kr (R.I.); khhkim@snu.ac.kr (K.H.K.); 3 Pondicherry Centre for Biological Science and Educational Trust, Kottakuppam 605104, Tamil Nadu, India; 4 Department of Animal Science and Technology, Konkuk University, Seoul 05029, Korea; rharhadl1995@snu.ac.kr; 5 Hanwoo Research Institute, National Institute of Animal Science, RDA, Pyeongchang 25342, Korea; jangsc@korea.kr; 6 Division of Animal Nutritional and Physiology, National Institute of Animal Sciences, Wanju 55365, Korea; chang4747@korea.kr; 7 Department of International Agricultural Technology, Graduate School of International Agricultural Technology, Seoul National University, Pyeongchang 25354, Korea; 8 Division of Animal Bioscience and Integrated Biotechnology, Gyeongsang National University, Jinju 52828, Korea

**Keywords:** lipogenesis, *GPAT1*, *SNAP23*, fatty acid composition, Hanwoo steer

## Abstract

**Simple Summary:**

Intramuscular fat or fatty acids content was regarded as a quality index of meat as it increases meat tenderness and flavor. In Korea, commercial Hanwoo farms have been supplying concentrate feeds that are 2–3% higher in crude protein (CP) content rather than conventional feeds throughout the entire program and have tried to shorten the feeding period to less than 28 months of age. This has led to a probable stability in lean-to-fat ratio in Hanwoo steers from 19 to 21 months of age. This stability could be linked to the regulation of the gene expression mediating lipogenesis during this phase. However, there is a lack of data regarding the effects of the protein level in isoenergetic concentrate diets on transcriptional activity of such genes at this phase. The conventional feeding program and the current one with relatively higher CP content were compared in two groups of twenty Hanwoo steers. Results showed that higher CP endorsement during the growing phase increased the expression of intramuscular *PPARα* (*p* < 0.1) and *LPL* (*p* < 0.05) and decreased the genes, such as *VLCAD* (*p* < 0.01), *GPAT1* (*p* = 0.001), and *DGAT2* (*p* = 0.016), which are involved in lipogenesis and fatty acid esterification. This may result in a relatively lower lipid turnover which could be responsible for shortening the feeding period.

**Abstract:**

The present study evaluated the influence of dietary protein level on growth performance, fatty acid composition, and the expression of lipid metabolic genes in intramuscular adipose tissues from 18- to 23-month-old Hanwoo steers, representing the switching point of the lean-to-fat ratio. Forty steers with an initial live weight of 486 ± 37 kg were assigned to one of two treatment groups fed either a concentrate diet with 14.5% CP and or with 17% CP for 6 months. Biopsy samples of intramuscular tissue were collected to analyze the fatty acid composition and gene expression at 23 months of age. Throughout the entire experimental period, all steers were restrained twice daily to allow individual feeding. Growth performance, blood metabolites, and carcass traits, according to ultrasonic measurements, were not affected by the experimental diets. The high-protein diet significantly increased the expression of intramuscular *PPARα* (*p* < 0.1) and *LPL* (*p* < 0.05) but did not affect genes involved in fatty acid uptake (*CD36* and *FABP4*) nor lipogenesis (*ACACA*, *FASN*, and *SCD*). In addition, it downregulated intramuscular *VLCAD* (*p* < 0.01) related to lipogenesis but also *GPAT1* (*p* = 0.001), *DGAT2* (*p* = 0.016), and *SNAP23* (*p* = 0.057), which are involved in fatty acid esterification and adipocyte size. Hanwoo steers fed a high-protein diet at 18–23 months of age resulted in a relatively lower lipid turnover rate than steers fed a low-protein diet, which could be responsible for shortening the feeding period.

## 1. Introduction

In the Korean beef cattle (Hanwoo) grading system, intramuscular fat (IMF) content of the *Longissimus dorsi* is a key quality factor, and the current grading system (1++, 1+, 1, 2, 3) has been implemented since 2004 [1]. Specific feeding strategies after castration at about 7 months of age have been developed for Hanwoo steers until 30 months of age [2,3]. The conventional feeding program in Korea is based on restricted feeding of concentrates but a gradual increase in the dietary concentrate to forage ratio from 50% to 90%, which supplies sufficient energy for stimulating fat deposition during the 17-month fattening phase. Forage is usually provided *ad libitum* during the growth phase (7–13 months) and gradually decreases from 30% to 10% of total dry matter (DM) intake from the early (14–21 months) to late fattening (22–30 months) phases, respectively. The total digestible nutrient (TDN) content of concentrate feeds has gradually been increased from 70% to 74% DM, and crude protein (CP) content has gradually been decreased from 16% to 12% DM [3,4]. However, commercial farms have been supplying concentrate feeds that are 2–3% higher in CP content rather than conventional feeds throughout the entire program and have tried to shorten the feeding period to less than 28 months of age. Positive results on growth performance and carcass characteristics have been obtained for Hanwoo steers by shortening the feeding period, attributed not only to a 2.5% increase in CP content but also to an increase in TDN content compared to conventional diets [5].

In a comparative slaughter study, Hanwoo steers recorded their highest daily gain and the switching point of the ratio of daily retained energy to daily lean body weight gain at 19–21 months of age; thereafter, the retained energy ratio dramatically increased until 30 months of age [6]. This indicates that the lean-to-fat ratio is relatively stable due to a preferential increase in protein from 19 to 21 months of age. It can be assumed that regulation of the gene expression mediating lipogenesis during this phase would lead to relatively stable changes. Gene expression detected by quantitative real-time polymerase chain reaction using five consecutive biopsy samples at 2, 7, 12, 20, and 25 months of age revealed that the inflection point and peak expression of many genes associated with adipogenesis and lipogenesis were observed at 20–25 months of age [7]. Furthermore, studies have also demonstrated the roles of different genes in intramuscular fatty acid synthesis and growth performance in different cattle breeds [8,9]. 

However, there is a lack of data regarding the effects of the protein level in isoenergetic concentrate diets on transcriptional activity of genes related to fatty acid biosynthesis and fatty acid composition during the growth stage, which represents the switching point of the lean-to-fat ratio in Hanwoo steers. Therefore, the present study evaluated growth performance, blood metabolites, fatty acid composition, and the expression of 14 genes involved in lipid metabolism in intramuscular adipose tissues of Hanwoo steers fed isoenergetic concentrate diets with different protein levels for 6 months from 18 to 23 months of age. 

## 2. Materials and Methods

### 2.1. Experimental Design, Animals, and Diet

All experiments were carried out at the animal farm of Seoul National University (Pyeongchang, Korea). Experiments were carried out according to the Guidelines for the Care and Use of Experimental Animals of Seoul National University (SNU-171211–1). Forty Hanwoo steers were selected from steers that had been reared at a farm with the same concentrate and hay according to a conventional feeding program. After a 1-month adaptation to the new pens, steers with an initial live weight of 486 ± 37 kg (average age 18 months) were blocked by body weight (BW) and randomly allocated to two experimental groups (five steers/pen and four pens/treatment) fed either of the concentrate diets with 14.5% CP (LCP) or 17% CP (HCP) for 6 months. The concentrates were formulated to provide 75% TDN at equal metabolizable energy on a DM basis (Table 1). Steers in a pen were restrained in self-locking stanchions twice daily (0800 and 1800 h) for about 1 h to allow them to consume the concentrate and ryegrass hay individually. They were given full access to water and a mineral block *ad libitum* in the pen, and the monthly live BW of the steers was measured from the beginning of the experiment and used to calculate the average daily gain (ADG) during the experimental period. Daily feed refusal was noted to estimate the dry matter intake (DMI) and the feed conversion ratio (DMI/ADG).

### 2.2. Carcass Evaluation, Blood Collection, and Analysis

After 180 day of feeding, the non-invasive and real-time assessment of the carcass in the live Hanwoo steers (23 months) were performed by ultrasonic scanning using Super-eye meat (FHK Co., Ltd., Tokyo, Japan) equipped with linear probe (2 MHz frequency: 27 × 147) between the 13th rib and lumbar vertebrae on the left side [10]. The estimates of back fat thickness, rib eye area, yield grade, marbling score, and quality grade were obtained using the ultrasound image.

One hour before the morning feeding on day 180 of the experiment, blood samples were collected from the jugular vein of two animals in each pen using a syringe (18G) and transferred to anticoagulant-free 6 mL yellow-capped BD Vacutainers^®^ in an icebox. Serum was separated from the blood by centrifugation at 2500× *g* for 15 min at 4 °C (ScanSpeed 1580R, Labogene, Seoul, Korea) and transferred to 2 mL microtubes for storage at −80 °C until further analysis. Biochemical parameters, including total cholesterol, glucose, triglycerides, and non-esterified fatty acids, were analyzed using an automatic analyzer (BS-400, Mindray, Beijing, China). 

### 2.3. Tissue Biopsies, RNA Extraction, and Real-Time Quantitative PCR

After 180 days of feeding, tissue samples (2 g/head) from eight animals in each group were collected by biopsy from the left-side rear of the third lumbar vertebra [11] using a spring-loaded biopsy instrument (Biotech, Karlova Ves, Slovakia), under intramuscular sedation (Xylazine 20 Inj., 20 mg/head, KEPRO B.V, Deventer, The Netherlands) and a line block (10 mL/head, 2% lidocaine injection, Cheil Pharma, Seoul, Korea) injection of local anesthesia. The harvested tissue samples were snap-frozen in liquid nitrogen and stored at −80 °C until further processing. The respective animals were intramuscularly injected with Procaine Penicillin G injection (4500 IU/kg, G.C. GPS Inj., Green Cross Veterinary Products, Seoul, Korea) immediately after collecting the intramuscular tissue. Then, for another 3 days, the animals were intramuscularly administrated 3 mg/kg Ketoprofen (New-Procop Inj., Shinil Biogen, Yesan-gun, Korea).

Total RNA was isolated from tissues using the RNeasy Lipid Tissue Mini Kit (Qiagen, Hilden, Germany) according to the manufacturer’s instructions. The purity and concentration of the isolated RNA were determined using a NanoDrop™ 2000/2000c spectrophotometer (Thermo Fisher Scientific, Waltham, MA, USA). The integrity of isolated RNA was evaluated by visualizing the 28S and 18S bands using eco dye-stained (Biofact) agarose gel electrophoresis. The cDNA synthesis (1 μg) from the isolated RNA was performed using the QuantiTect Reverse Transcription Kit (Qiagen) according to the manufacturer’s instructions. In the current study, 14 genes (Table 2) were investigated using real-time qPCR with SYBR Green real-time-PCR Master Mix (Bioneer, Seoul, Korea) and the CFX96 Touch™ Real-Time PCR Detection System (Bio-Rad Laboratories, Inc., Hercules, CA, USA). The 20-µL reaction mixture contained 30 ng cDNA, 10 µL SYBR Green Master Mix, and 1.0 µL each 10 μM primer. The PCR conditions were 95 °C for 15 min, followed by 40 cycles at 94 °C for 15 s, annealing temperature for 30 s, and 72 °C for 30 s. All primers were designed using the Primer-BLAST [12] tool based on the National Center for Biotechnology Information published sequences (www.ncbi.nlm.nih.gov, accessed on 5 January 2018). The 2^−ΔΔCT^ method was used to determine relative-fold changes [13], and all data were normalized based on the β-actin housekeeping gene.

### 2.4. Chemical and Fatty Acid Analyses

The feed samples were dried in a forced-air oven at 65 ℃ for 72 h to estimate DM content and then ground to pass through a 1 mm screen (Model 4; Thomas Scientific, Swedesboro, NJ, USA). The feed samples were then analyzed for nutrient compositions, such as CP (Method 990.03 [14]), ash (Method 942.05 [14]), and ether extract (Method 960.39 [14]). Neutral detergent fiber content was assayed with heat-stable amylase (without sodium sulfite) and expressed exclusively as residual ash (aNDFom) using the method of Van Soest et al. [15]. The acid detergent fiber content, excluding residual ash (ADFom), was determined according to Van Soest [16]. 

The fatty acid composition of the biopsied intramuscular tissues was determined according to the method described by O’Fallon et al. [17]. A commercial 37-component fatty acid methyl ester (FAME) standard mixture (CRM47885) and the internal standard tridecanoic acid (C13:0) were obtained from Supelco Co. (Belafonte, PA, USA). The extracted fatty acids (1 µL) were injected with a split ratio of 30:1 into an Agilent 7890B GC system (Agilent Technologies, Santa Clara, CA, USA) equipped with a flame ionization detector (FID) and a 100  m × 0.25 mm × 0.20 μm SP-2560 biscyanopropyl polysiloxane capillary column (Cat. No: 24056, Supelco, Sigma-Aldrich, St. Louis, MO, USA). The inlet and detector temperatures were maintained at 250 °C and 260 °C, respectively. The helium carrier gas was set to a flow rate of 1.18 mL/ min, and the initial oven temperature was set at 100 °C. The oven temperature was held constant at the initial temperature for 5 min and then increased by 4 °C /min to a final temperature of 240 °C, which was held for 14 min. The fatty acid contents were quantified after normalization with the internal standard using the fatty acid standards described by the AOCS Official Method Ce 1j-07 [18]. Theoretical FID correction factors reported in the AOCS Official Method Ce 1 h-05 [19] were applied to individual FAMEs. The concentrations of individual fatty acids are expressed in terms of total FAMEs instead of tissue content.

### 2.5. Statistical Analysis

Data were completely randomized, with steer as the experimental unit, using the MIXED procedure in SAS software, version 9.4 (SAS Institute, Cary, NC, USA). The fixed effects in the model included the dietary treatments. Animals within a treatment group were considered a random effect. Appropriate covariance structures were chosen based on Akaike’s information criterion. Means were calculated using the LSMEANS statement, and treatment differences were considered significant at *p* < 0.05. Near significant trends were considered present at 0.05 < *p* < 0.10 [20]. Pearson’s correlation was used to test the correlations between fatty acid composition and genes, and among genes using the corr.test function in the ‘psych’ package of R-software, version 4.0.3 (The R Foundation for Statistical Computing, Vienna, Austria). 

## 3. Results 

### 3.1. Feed Intake, Daily Gain, Blood Metabolites, and Fatty Acid Composition of Biopsy Tissues

Feeding a high-protein concentrate during 18- to 23-month age period did not affect (*p* > 0.05) feed intake, ADG, blood metabolites, nor the ultrasonic evaluation of carcass traits in Hanwoo steers (Table 3 and Table 4). However, a trend toward increased total fatty acids (*p* = 0.07) and oleic acid (*p* = 0.09) proportion and a significant increase in eicosenoic acid (*p* < 0.01) proportion in intramuscular tissue were noted (Table 5). 

### 3.2. Intramuscular Lipid Metabolic Genes Expression and Their Associations 

**Figure 1:**
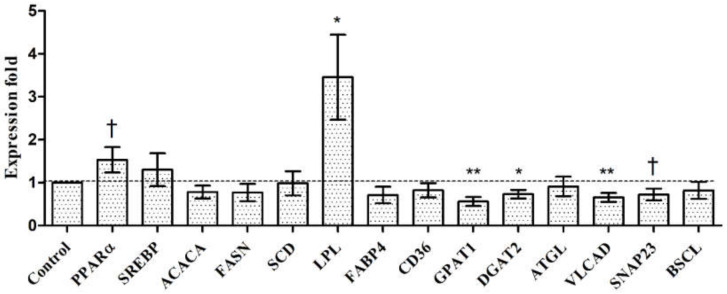
Effect of 6 months of an altered dietary protein level on the relative expression levels of genes in intramuscular tissue of Hanwoo steers sampled by biopsy at 23 months of age. ^†^ *p* < 0.1; *p < 0.05; ** < 0.01. Values are the least-squares means with the standard error (*n* = 8).

Figure 1 illustrates the effects of fattening diets with varying protein concentrations at 18–23 months of age on the expression patterns of lipid metabolic genes in intramuscular tissues. The dietary treatments had no effects (*p* > 0.05) on expression of genes, such as sterol regulatory element-binding protein (*SREBP*), fatty acid synthase (*FASN*), acetyl-CoA carboxylase (*ACACA*), or stearoyl-CoA desaturase (*SCD*), Berardinelli-Seip congenital lipodystrophy2-seipin (*BSCL)*, and adipose triglyceride lipase (*ATGL*). The high-protein diet upregulated only intramuscular expression of peroxisome proliferator-activated receptor (*PPARα*) by 1.5-fold (*p* = 0.09) and lipoprotein lipase (*LPL*) by 3.5-fold (*p* < 0.05). However, a downregulation in glycerol-3-phosphate acyltransferase (*GPAT1*) (*p* < 0.005)*,* diacylglycerol acyltransferase-2 (*DGAT2*) (*p* < 0.05), very long-chain acyl-CoA dehydrogenase (*VLCAD*) (*p* < 0.005), and synaptosome-associated protein 23 (*SNAP23*) (*p* = 0.057) genes were noted. 

Pearson’s correlation analysis revealed a wide range of associations among different genes expressed (Supplementary Table S1). Most notably, *PPARα* expression was positively correlated with *LPL* (*p* < 0.05) and negatively with *VLCAD* (*p* < 0.05). Likewise, *SCD* expression was positively correlated with *DGAT2* (*p* < 0.05), *FASN* (*p* < 0.001), and *ACACA* expression (*p* < 0.005). Associations between genes and fatty acid composition are showed in Supplementary Table S2. Oleic acid content was positively and negatively associated with the expression level of *LPL* (*p* = 0.060) and *FABP4* (*p* = 0.060), respectively. A strong (*p* < 0.05) negative association of *FASN* and *ACACA* with alpha linoleic acid content was also noted. 

## 4. Discussion

The observed effect of dietary protein concentration on growth performance of 18–23 months old Hanwoo steers are consistent with a previous study [21] that assessed the effects of different levels of dietary protein in 13- to 18-month-old finishing steers. Kamiya et al. [22] also did not find an effect of high CP level on the daily gain of Holstein steers. In addition, several studies have reported that diets containing more or less protein than the recommended amount do not lead to significant differences in marbling or IMF content [23,24]. Lee et al. [25] showed that diets with higher CP and non-degraded protein intake levels do not affect growth performance but tend to improve the carcass quality of Hanwoo steers. On the other hand, high total fatty acids and oleic acid contents are positively correlated with flavor [26], customer preference over imported beef [27], and favorable health characteristics for consumers [28,29]. Rats that eat Hanwoo beef fat show high feed intake but reduced lipogenic enzyme activities and increased high-density lipoprotein cholesterol, indicating a lower risk for cardiovascular disease compared to consuming Angus beef fat [30].

Peroxisome proliferator-activated receptors (*PPAR*s) are fatty acids regulated transcription factors that control lipid metabolism [31]. Among the *PPAR* isoforms, *PPARα* binds to the *PPAR* response element in the *LPL* gene promoter and enhances *LPL* gene expression in adipose tissue [32] and human skeletal muscle [33]. The increased expression of *LPL* gene and high content of long-chain mono- and poly-unsaturated fatty acids in the HCP group may be supported by the functional role of *PPARα* binding to fatty acids with a general preference for long-chain unsaturated fatty acids [34]. This was further supported by the strong positive association between *LPL* and *PPARα* expression in the current experiment. In addition, adipose tissue *LPL* of young rats [35] and yaks [36] is downregulated by a low-protein diet and stimulated by a high-protein diet. *LPL* is the rate-limiting enzyme that hydrolyzes plasma triglycerides and plays key roles in lipoprotein metabolism through the efficient transfer of energy in the form of lipid from sites of synthesis to sites of storage or utilization [37]. In this sense, upregulation of *LPL* expression caused by the increase in dietary protein concentration at 18–23 months of age may facilitate uptake of fatty acids because Hanwoo steers typically start to retain energy in the form of fat into vascular, subcutaneous, and intramuscular tissues between 19 and 21 months of age [6]. Okumura et al. [38] observed that an additional fattening period of 6 months in 24- to 30-month-old Japanese black steers increases the rate of intramuscular fat deposition. Jeong et al. [9] reported a strong correlation between *LPL* mRNA abundance and IMF content in Hanwoo steers and suggested that *LPL* is a genetic marker for IMF deposition. In addition, the observed positive association between *LPL* and oleic acid (*p* < 0.005) in this experiment suggested that the expression of LPL might be responsible for increase in oleic acid content in intramuscular tissue.

Berardinelli-Seip congenital lipodystrophy2-seipin (*BSCL)* is an endoplasmic reticulum (ER) membrane protein involved in regulation of lipid droplet biogenesis. Payne et al. [39] strongly suggested that downregulation of *BSCL* causes a defect in lipid droplet morphology formation, indicating that it is crucial for normal adipogenesis of adipocytes. That study also showed that cells lacking *BSCL* fail to induce the expression of *SREBP* and the lipogenic enzymes *GPAT1* and *DGAT2*; our results are consistent with those findings. *GPAT1* is involved in the first step of triglyceride synthesis via acylation of glycerol 3-phosphate and direct incorporation of exogenous fatty acids into triglycerides rather than phospholipids [40]. Multiple regression analyses between IMF contents and the abundance of genes responsible for fat deposition and fat removal have revealed that *GPAT1* is a candidate gene for increasing IMF deposition in Hanwoo [9]. The *DGAT2* gene is an important contributor to triglyceride synthesis and storage and increases the total proportion of polyunsaturated fatty acids to saturated fatty acids within the adipocyte. Our results are similar to a previous study that reported downregulation of *DGAT2* expression in cattle fed a high-protein diet with fat [41]. Despite the observed downregulation of *GPAT1* and *DGAT2* genes involved in fatty acid esterification, the intramuscular fatty acid and oleic acid contents increased in response to the high-protein diet in this experiment. *DGAT* activity is not the only mechanism for triglyceride synthesis, as adequate triglyceride biosynthesis can be sustained by other enzymes via multiple mechanisms even under downregulation of *DGAT2* [42]. Yu et al. [43] demonstrated that *DGAT2* plays an important role in energy homeostasis through rigorous post-transcriptional gene expression in adipocytes. It is unclear if this type of triglyceride biosynthesis plays a significant role in mammalian cells, as another major triglyceride bypassing *DGAT* has been identified in yeast [44]. The interaction between fatty acid translocase (CD36) and long-chain fatty acids is important for the absorption and storage of dietary lipids [9]. Adipocyte fatty acid-binding protein 4 (*FABP4*) directly interacts with hormone-sensitive lipase, which is the first step in an organized lipid transfer process that leads to an increase in fatty acid hydrolysis. However, in the absence of the interaction, fatty acids are not efficiently released from the adipocyte and accumulate intracellularly [45,46]. These genes associated with fatty acid uptake are not affected by increasing the dietary protein concentration in this experiment. Stearoyl-CoA desaturase (SCD) is a membrane-bound enzyme that synthesize monounsaturated fatty acids from saturated fatty acids [47]. Although a strong positive association was noted between the expression of *DGAT2* and *SCD* in this experiment, they did not account for the physical interaction between both genes during fatty acid monounsaturation [48] and the incorporation of endogenously synthesized monounsaturated fatty acids into triglycerides [49]. Archibeque et al. [50] reported that total unsaturated fatty acid and oleic acid contents do not result from greater *SCD* gene expression in the adipose tissues of beef steer. In the present experiment, unchanged or suppressed genes related to fatty acid uptake, lipogenesis, and fatty acid esterification appeared to originate from the effects of dietary protein level and not from age-associated changes in lipogenesis of 18- to 23-month-old Hanwoo steers. A peak in expression of many genes associated with adipogenesis and lipogenesis is detected at 25 months of age; thereafter, cattle are beginning to deposit a substantial amount of IMF [7]. Additional increases in the expression of those genes are observed during the finishing phase [51,52]. 

Very long-chain acyl-CoA dehydrogenase (*VLCAD*) catalyzes the initial step in the mitochondrial β-oxidation of long-chain fatty acids. Adiposity, or the amount of triglyceride stored in adipocytes, is fundamentally a net result of lipogenesis and lipolysis. The observed downregulation of *VLCAD* by high protein diet in the current study indicated that the lipid turnover rate, i.e., the balance between the synthesis and degradation of triglycerides, was relatively lower in steers fed the high-protein diet than in those fed the low-protein diet, which could be responsible for the carryover effect on high marbling in later life. Jeong et al. [9] reported that the expression of the fat removal gene *VLCAD* is negatively correlated with IMF content. The negative correlation between *PPARα* and *VLCAD* can be explained by muscle-specific upregulation of *PPARα* in *VLCAD*-deficient mice [53]. 

The abundance of *SNAP23* transcripts is positively correlated with adipocyte size [54]. Therefore, downregulation of intramuscular *SNAP23* expression, mediated by the high-protein diet, may have contributed to reduced adipocyte size and increased fineness of the carcass marbling texture. Vierck et al. [55] reported that coarsely marbled steaks contain larger adipocytes than those of finely marbled steaks. This may serve as a pointer for future investigations to elucidate the mechanism of synthesis of fine marbling because Korean consumers prefer fine marbling and the quality grading system changes based on fineness. Although growth performance and final carcass traits after 23 months of age could not be included in this study, indirect identification of the functional links between gene regulation and adipose tissue occurred at a particular developmental stage for 6 months, which was made possible by the developmental processes related to the data [3] on the change in the lean-to-fat ratio or net energy content during daily gain of Hanwoo steers. 

## 5. Conclusions

A high-protein diet fed to 18- to 23-month-old steers altered the switching point of the lean-to-fat ratio and could be responsible for less lipogenesis (*GPAT1* and *DGAT2*) and lipolysis (*VLCAD*) compared to a low-protein diet. These data reveal a relatively low lipid turnover rate, which could be responsible for shortening the feeding period. Furthermore, Hanwoo steers fed a high-protein diet during this period showed increased intramuscular fatty acid content, oleic acid, and fineness in the marbling texture during later life by downregulating *SNAP23*.

## Figures and Tables

**Table 1:** Ingredients and nutrient composition of the diets fed to steers during the experimental period.

Items	Concentrates		Ryegrass Hay
	LCP	HCP	
*Ingredient (% DM)*			
Broken corn	1.36	1.02	
Wheat bran	4.44	5.56	
Soy bean hull	7.08	2.12	
Urea	0.45	0.58	
NaCl	0.20	0.20	
Molasses	3.00	3.00	
Baking soda	1.07	0.94	
Steam flaked corn	22.00	22.00	
Ammonium chloride	0.15	0.15	
Corn flour	1.72	0.05	
Extruded palm seed	4.24	7.52	
CMS	1.50	1.50	
Wheat flour	20.02	20.00	
DDGS	10.00	13.00	
Corn gluten feed	20.00	19.92	
Limestone	2.34	2.00	
Amaferm ^1^	0.01	0.01	
Palm oil	0.20	0.20	
Mineral/Vitamin premix ^2^	0.23	0.23	
*Nutrient composition (% DM)*			
DM (%)	89.0	89.0	90.0
Ash	6.6	6.4	4.4
Crude Protein	14.5	17.0	5.7
Ether extract	3.4	3.7	1.2
NDF	22.0	22.0	67.1
ADF	8.4	8.1	40.8
TDN	75.1	75.2	-

LCP, low crude protein; HCP, high crude protein; CMS, condensed molasses solubles; DDGS, distiller’s dried grain with solubles; NDF, neutral detergent fiber; ADF, acid detergent fiber; TDN, total digestible nutrients. ^1^ Fermentation extract of *Aspergillus oryzae* (Biozyme Enterprises Inc., St. Joseph, MO, USA). ^2^ Provided the following nutrients per kg additive (Grobic-DC, Bayer Health Care, Leverkusen, Germany): Vit. A, 2,650,000 IU; Vit. D, 3, 530,000 IU; Vit. E, 1050 IU; Niacin, 10,000 mg; Mn, 4400 mg; Zn, 4400 mg; Fe, 13,200 mg; Cu, 2200 mg; I, 440 mg; Co, 440 mg.

**Table 2:** Gene names, GenBank accession numbers, sequences, and amplicon sizes of the *Bos taurus* primers used in the real-time quantitative PCR analysis.

Gene	NCBI Acc. No.	Primer Name	Primer Sequence (5′–3′)	Product Size (bp)
*Transcription factors:*				
Peroxisome proliferator activated receptors	NM_001034036.1	PPAR FP	CAATGGAGATGGTGGACACA	95
		PPAR RP	TTGTAGGAAGTCTGCCGAGAG	
Sterol regulatory element-binding proteins	NM_001113302.1	SREBP FP	GAGCCACCCTTCAACGAA	88
		SREBP RP	TGTCTTCTATGTCGGTCAGCA	
*Lipogenesis*:				
Acetyl-CoA carboxylase	NM_174224.2	ACACA FP	CGCTCGGTGATTGAAGAGAA	117
		ACACA RP	CGTCATGTGGACGATGGAAT	
Fatty acid synthase	NM_001012669.1	FASN FP	ATCGAGTGCATCAGGCAAGT	92
		FASN RP	TGTGAGCACATCTCGAAAGCCA	
Stearoyl-CoA desaturase	NM_173959.4	SCD FP	TTATTCCGTTATGCCCTTGG	83
		SCD RP	TTGTCATAAGGGCGGTATCC	
*Fatty acid uptake:*				
Lipoprotein lipase	NM_001075120.1	LPL FP	CTCAGGACTCCCGAAGACAC	98
		LPL RP	GTTTTGCTGCTGTGGTTGAA	
Fatty acid binding protein 4	NM_174314.2	FABP4 FP	GGATGATAAGATGGTGCTGGA	80
		FABP4 RP	ATCCCTTGGCTTATGCTCTCT	
Fatty acid translocase (CD36)	NM_174010.3	CD36 FP	GGTCCTTACACATACAGAGTTCG	115
		CD36 RP	ATAGCGAGGGTTCAAAGATGG	
*Fatty acid esterification:*				
Glycerol-3-phosphate acyltransferase-1	NM_001012282.1	GPAT1 FP	TGTGCTATCTGCTCTCCAATG	116
		GPAT1 RP	CTCCGCCACTATAAGAATG	
Diacylglycerol acyltransferase-2	NM_205793.2	DGAT2 FP	CATTGCCGTGCTCTACTTCA	86
		DGAT2 RP	AGTTTCGGACCCACTGTGAC	
*Lipolysis*:				
Adipose triglyceride lipase	NM_001046005.2	ATGL FP	TGACCACACTCTCCAACA	100
		ATGL RP	AAGCGGATGGTGAAGGA	
Very long chain acyl-CoA dehydrogenase	U30817.1	VLCAD FP	TCTTCGAGGGGACAAATGAC	116
		VLCAD RP	AGCATTCCCAAAAGGGTTCT	
*Adipocyte size:*				
Synaptosome-associated protein 23	BT030678.1	SNAP23 FP	GGAGGGGAGGCAAGAGATAA	148
		SNAP23 RP	AAACCAAGCACTGGCCTAAA	
Berardinelli-Seip congenital lipodystrophy2-seipin	BC105396.1	BSCL2 FP	CGAAAGGTCTCTGCCCATC	140
		BSCL2 RP	GTTTTCTCCTCCTCGGACAG	
*Housekeeping*:				
Beta-actin	BC142413.1	β-Actin FP	GTCCACCTTCCAGCAGATGT	90
		β -Actin RP	CAGTCCGCCTAGAAGCATTT	

PCR, polymerase chain reaction.

**Table 3:** Effects of dietary protein level on feed intake, growth performance (*n* = 20), and serum metabolites (*n* = 8) in Hanwoo steers.

Item	LCP	HCP	SEM	*p*-Value
DMI (kg/d)	9.5	9.5	0.02	0.993
Concentrate (% DMI)	81.6	82.0	0.17	0.111
Rye grass hay (% DMI)	17.5	17.1	0.31	0.379
Body weight (kg)				
Initial	484.6	487.5	9.04	0.822
Final	621.2	626.7	12.44	0.756
Average daily gain (kg/d)	0.76	0.77	0.04	0.815
Feed conversion ratio ^1^	13.3	12.9	0.70	0.646
*Serum metabolites*				
Cholesterol, mg/dL	134.9	155.4	15.90	0.377
Triglycerides, mg/dL	17.3	20.5	1.47	0.141
Glucose, mg/dL	40.5	38.9	2.67	0.674
NEFA, mmol/L	0.1	0.1	0.01	0.226

LCP, low crude protein; HCP, high crude protein; NEFA, non-esterified fatty acids; SEM, standard error of the mean. ^1^ Feed conversion ratio = average daily intake/average daily gain.

**Table 4:** Effect of dietary protein level during the growing phase on carcass characteristics of Hanwoo steers evaluated using ultrasonic scanning at 23 months of age (*n* = 20).

Item	LCP	HCP	SEM	*p*-Value
Back fat thickness (mm)	7.08	6.90	0.48	0.799
Rib eye area (cm^2^)	82.13	81.83	1.20	0.860
Yield grade (A:B:C, head) ^1^	17:3:0	18:2:0		
Yield grade score ^2^	2.85	2.90	0.08	0.643
Marbling score ^3^	3.00	2.95	0.24	0.883
Quality grade (1^+^:1:2:3, head) ^4^	0:7:12:1	1:3:16:0		
Quality grade score ^5^	2.35	2.25	0.13	0.582

LCP, low crude protein; HCP, high crude protein; SEM, standard error of the mean. ^1^ Carcass yield grades range from C (low yield) to A (high yield). ^2^ Yield grade score: A = 3, B = 2, and C = 1. ^3^ Marbling score ranges from 1 to 9, with higher numbers indicating better quality (1 = devoid, 9 = abundant). ^4^ Quality grades range from 3 (low quality) to 1^+^ (high quality). ^5^ Quality grade score: 1^+^ = 4, 1 = 3, 2 = 2, and 3 = 1.

**Table 5:** Effect of dietary protein level at growing stage on fatty acid composition in the intramuscular tissue of Hanwoo steers sampled by biopsy at 23 months of age (*n* = 8).

Fatty Acid (mg/100 g FAME)	LCP	HCP	SEM	*p*-Value
Myristic acid (C14:0)	3571	3476	283.7	0.815
Palmitic acid (C16:0)	26339	26921	1213.7	0.639
Palmitoleic acid (C16:1n7)	4062	4382	495.8	0.656
Stearic acid (C18:0)	10123	10606	572.7	0.560
Oleic acid (cis 9 C18:1)	37192	39747	990.7	0.090
Linoleic acid (C18:2n6c)	2283	2861	393.3	0.316
Gamma-linolenic acid (C18:3n6)	34	34	2.5	0.816
Alpha linolenic acid (C18:3n3)	114	129	7.9	0.218
Eicosenoic acid (C20:1n9)	147	217	19.0	0.008
Arachidonic acid (C20:4n6)	424	672	225.3	0.449
Others ^1^	5843	6169	693.2	0.621
SFA ^2^	41758	42705	1482.2	0.659
MUFA ^3^	45201	48442	1261.0	0.091
PUFA ^4^	3173	4067	666.3	0.359
Total fatty acids (mg/100 g FAME)	90132	95214	1842.4	0.071

SFA, saturated fatty acids; MUFA, monounsaturated fatty acids; PUFA, polyunsaturated fatty acids; FAME, fatty acid methyl ester. ^1^ Others = C4:0 + C6:0 + C8:0 + C10:0 + C11:0 + C12:0 + C14:1 + C15:0 + C15:1 + C17:0 + C17:1 + C18:1n9t + C18:2n6t + C20:0 + C20:2n6 + C20:3n6 + C20:3n3 + C20:5n3 + C21:0 + C22:0 + C22:1n9 + C22:2n6 + C23:0+ C24:0+C24:1n9 + C22:6n3. ^2^ SFA = C10:0 + C11:0 + C12:0 + C14:0 + C15:0 + C16:0 + C17:0 + C18:0 + C20:0 + C21:0 +C22:0 + C24:0. ^3^ MUFA = C14:1n5 + C16:1n7 + C17:1n7 + C18:1n7 + C18:1n9 + C20:1n9 + C22:1n9 + C24:1n9. ^4^ PUFA = C18:2n6 + C18:2c9,t11 + C18:3n3 + C18:3n6 + C20:2n6 + C20:3n3 + C20:3n6 + C20:4n6 + C20:5n3 + C22:2n6 + C22:4n6 + C22:5n3 + C22:6n3.

## Data Availability

All data generated in this study are included in the manuscript and Supplementary Files.

## Supplementary Materials

The following are available online at https://www.mdpi.com/article/10.3390/ani11123378/s1, Table S1: Correlation coefficients for the expression of genes in intramuscular tissues; Table S2: Correlation coefficients for fatty acid composition and gene expression in intramuscular tissues.
