# Supplementary material for: Effects of Dietary Protein Concentration on Lipid Metabolism Gene Expression and Fatty Acid Composition in 18–23-Month-Old Hanwoo Steers
Source: Animals (Basel). 2021 Nov 25;11(12):3378. doi: 10.3390/ani11123378 (PMC8697893; doi:10.3390/ani11123378)
Supplement: Supplementary file 1 [file animals-11-03378-s001.zip › animals-1458797-supplementary.pdf]

Supplementary material

Effects of Dietary Protein Concentration on Lipid Metabolism Gene Expression and Fatty Acid Composition in 18–23-Month-Old Hanwoo Steers

Rajaraman Bharanidharan [1], Krishnaraj Thirugnanasambantham [2,3,†], Ridha Ibidhi [2], Geumhwi Bang [4], Sun Sik Jang [5], Youl Chang Baek [6], Kyoung Hoon Kim [2,7] and Yea Hwang Moon [8,*]

[1] Department of Agricultural Biotechnology, College of Agriculture and Life Sciences, Seoul National University, Seoul 08826, Korea; bharanidharan7@snu.ac.kr
[2] Department of Eco-friendly Livestock Science, Institute of Green Bio Science and Technology, Seoul National University, Pyeongchang 25354, Korea; thiru_dna@yahoo.co.in (K.T.); ridha@snu.ac.kr (R.I.); khhkim@snu.ac.kr (K.H.K.)
[3] Pondicherry Centre for Biological Science and Educational Trust, Kottakuppam 605104, Tamil Nadu, India
[4] Department of Animal Science and Technology, Konkuk University, Seoul 05029, Korea; rharhadl1995@snu.ac.kr
[5] Hanwoo Research Institute, National Institute of Animal Science, RDA, Pyeongchang 25342, Korea; jangsc@korea.kr
[6] Division of Animal Nutritional and Physiology, National Institute of Animal Sciences, Wanju 55365, Korea; chang4747@korea.kr
[7] Department of International Agricultural Technology, Graduate School of International Agricultural Technology, Seoul National University, Pyeongchang 25354, Korea
[8] Division of Animal Bioscience and Integrated Biotechnology, Gyeongsang National University, Jinju 52828, Korea
* Correspondence: yhmoon@gntech.ac.kr; Tel.: +82-55-772-3265
† These authors contributed equally to this work.

Table S1. Correlation coefficients for the expression of genes in intramuscular tissues.

Gene/Gene	*GPAT1*	*PPARα*	*DGAT2*	*CD36*	*VLCAD*	*FASN*	*ATGL*	*LPL*	*ACACA*	*SREBP*	*BSCL*	*FABP4*	*SCD*
SNAP23	-0.43†	-0.44†	0.39	0.63*	0.68	0.42	0.52*	0.20	0.50*	0.22	0.29	0.51*	0.30
SCD	-0.12	0.09	0.53*	0.14	0.06	0.89***	0.36	0.48†	0.73**	0.33	0.28	0.30	
FABP4	-0.52*	-0.47†	0.69**	0.10	0.58*	0.41	0.21	-0.17	0.46†	-0.20	-0.08		
BSCL	-0.04	0.02	0.09	0.45†	0.30	0.20	0.38	0.23	0.29	0.63*			
SREBP	0.46†	0.48†	-0.30	0.61*	-0.13	0.15	0.75**	0.66**	0.20				
ACACA	-0.33	-0.23	0.71**	0.22	0.30	0.90***	0.30	0.37					
LPL	0.22	0.56*	-0.17	0.47†	-0.10	0.42	0.63*						
ATGL	-0.03	0.36	0.00	0.72**	0.07	0.24							
FASN	-0.28	-0.17	0.69**	0.15	0.19								
VLCAD	-0.59*	-0.53*	0.53*	0.27									
CD36	0.15	0.28	-0.03										
DGAT2	-0.67**	-0.44†											
PPARα	0.59												

Correlation coefficients are across all treatments. Number of observations = 16. †P < 0.1; *P < 0.05; **P < 0.005; ***P < 0.001

Table S2. Correlation coefficients for fatty acid composition and gene expression in intramuscular tissues.

**Fatty acids/Gene**	***SNAP23***	***SCD***	***FABP4***	***BSCL***	***SREBP***	***ACACA***	***LPL***	***ATGL***	***FASN***	***VLCAD***	***CD36***	***DGAT2***	***PPARα***	***GPAT1***
Myristic acid (C14 :0)	-0.15	0.07	0.28	-0.49*	-0.46†	0.18	-0.26	-0.38	0.20	-0.15	-0.30	0.28	-0.12	0.02
Palmitic acid (C16 :0)	-0.16	0.01	-0.02	-0.57*	-0.50*	0.07	0.03	-0.41	0.13	-0.12	-0.21	0.08	0.11	0.06
Palmitoleic acid (C16 :1)	-0.18	0.04	-0.04	0.06	-0.10	0.17	-0.03	-0.05	0.11	0.07	-0.18	0.43	0.09	-0.33
Stearic acid (C18 :0)	0.34	0.15	-0.09	-0.24	0.03	0.18	0.38	0.14	0.18	0.03	0.35	-0.11	0.20	0.16
Oleic acid (cis 9 C18 :1)	-0.02	0.13	-0.43†	0.03	0.30	0.03	0.49†	0.12	0.05	-0.13	0.15	-0.26	0.40	0.29
Linoleic acid (C18 :2n6c)	-0.23	-0.19	-0.19	-0.21	-0.05	-0.33	0.13	0.10	-0.30	-0.05	-0.26	-0.20	0.15	-0.21
Gamma-Linolenic acid (C18 :3n6)	0.12	-0.22	-0.04	-0.20	-0.07	-0.39	0.03	0.09	-0.32	0.23	-0.10	-0.20	-0.11	-0.23
Alpha linolenic acid (C18 :3n3)	-0.28	-0.30	-0.18	-0.10	-0.01	-0.45†	-0.10	0.09	-0.52*	0.00	-0.16	-0.19	0.29	-0.08
Eicosenoic acid (C20:1n9)	-0.51*	-0.22	-0.56*	-0.13	0.20	-0.42	0.23	0.09	-0.42	-0.46†	-0.26	-0.48†	0.48†	0.22
Arachidonic acid (C20 :4n6)	-0.22	-0.20	-0.17	-0.25	-0.09	-0.30	0.12	0.04	-0.25	-0.08	-0.30	-0.20	0.03	-0.21
SFA	0.01	0.08	0.01	-0.51	-0.37	0.15	0.10	-0.27	0.19	-0.08	-0.04	0.07	0.11	0.11
MUFA	-0.11	0.14	-0.35	0.11	0.27	0.10	0.41	0.12	0.08	-0.08	0.07	-0.04	0.41	0.14
PUFA	-0.22	-0.20	-0.18	-0.24	-0.07	-0.33	0.13	0.08	-0.29	-0.05	-0.26	-0.20	0.11	-0.21
Total fatty acids (mg/100 g FAME)	-0.14	0.09	-0.29	-0.37	-0.11	0.07	0.39	-0.09	0.10	-0.13	-0.07	-0.05	0.39	0.10

Correlation coefficients are across all treatments. Number of observations = 16 †$P < 0.1$; *$P < 0.05$; **$P < 0.005$; ***$P < 0.001$.
